# The Effect of Reduced Oxygen Saturation on Retinal Microvascularization in COVID-19 Patients with Bilateral Pneumonia Based on Optical Coherence Tomography Study

**DOI:** 10.3390/jpm12111824

**Published:** 2022-11-02

**Authors:** Magdalena Kal, Mateusz Winiarczyk, Jerzy Mackiewicz, Dominik Odrobina, Elżbieta Cieśla, Bernadetta Płatkowska-Adamska, Michał Biskup, Paweł Pabjan, Dorota Zarębska-Michaluk

**Affiliations:** 1 Collegium Medicum of Jan Kochanowski University in Kielce, 25-369 Kielce, Poland; 2 Ophthalmic Clinic of the Voivodeship Hospital in Kielce, 25-736 Kielce, Poland; 3 Department of Vitreoretinal Surgery, Medical University of Lublin, 20-079 Lublin, Poland; 4 Insitute of Medical Science of Jan Kochanowski University in Kielce, 25-369 Kielce, Poland; 5 Department of Infectious Disease, Provincial Hospital in Kielce, 25-317 Kielce, Poland

**Keywords:** retinal microvasculature, OCT, OCTA, COVID-19, SARS-CoV-2, optical coherence tomography angiography, vessel density

## Abstract

The aim of the study was to evaluate changes in the retinal thickness and microvasculature based on optical coherence tomography (OCT) depending on baseline oxygen saturation (SpO2) in patients hospitalized due to COVID-19 bilateral pneumonia. The prospective study was carried out among 62 patients with COVID-19 pneumonia who underwent ophthalmic examination after hospital discharge. They were divided into three groups depending on the oxygen saturation (SpO2) on admission: ≤90% (group 1), >90% and ≤95% (group 2), and >95% (group 3). The following parameters were assessed in the ophthalmological examination and correlated with the baseline SpO2: ganglion cell layer (GCL), the retinal nerve fiber layer (RNFL) in the macular area, RNFL in the peripapillary area, the foveal avascular zone (FAZ) in superficial capillary plexus (SCP) and deep capillary plexus (DCP), vessel density (VD) in SCP, in DCP, and in the choriocapillaris plexus (CC). Baseline saturation ≤90% in COVID-19 patients caused a decrease of VD in some areas of SCP and DCP and an increase in FAZ area in SCP and DCP. In the group of patients with SpO2 ≤ 90% statistically significant thinning of the retina in the inner superior ring (ISR) (*p* = 0.029), the inner temporal ring (ITR) (*p* = 0.34), the outer superior ring (OSR) (*p* = 0.012), and the outer temporal ring (OTR) (*p*= 0.004)] was observed. The statistically significant thickening of RNFL optic disc and thinning of RNFL retina in some macular areas in patients with SpO2 ≤ 90% were reported. The size of FAZ area in SCP and vessel density were significantly greater in some areas of SCP, DCP, and CC in patients with SpO2 ≤ 90% (*p* = 0.025). Baseline oxygen saturation ≤90% has been found to influence the ocular parameters of OCT in COVID-19 patients. We noticed a widened FAZ zone in SCP and increased VD in some regions of the retina and choroid as a response to systemic hypoxia.

## 1. Introduction

Since the end of 2019, the severe acute respiratory syndrome coronavirus 2 (SARS-CoV-2) has spread worldwide, resulting in the pandemic announced by World Health Organization (WHO) on 11 March 2020. SARS-CoV-2 is transmitted by respiratory droplets, close contact with the infected person, or aerosols. The disease it causes, termed coronavirus disease 2019 (COVID-19), is mild or even asymptomatic in most of the patients affected, while about a fifth of them experience a severe course [1,2,3,4]. According to the literature data, up to 20% of COVID-19 develop severe ground-glass bilateral pneumonia, which can lead to acute respiratory distress syndrome and multi-organ failure due to the cytokine storm [5]. The risk of a severe course of COVID-19 and death is documented in elderly patients and those with comorbidities such as diabetes, obesity, chronic pulmonary diseases, chronic heart failure, malignant diseases, chronic heart failure, chronic respiratory failure, chronic renal impairment, and immunodeficiency [6,7]. A worse prognosis is also associated with coagulation disorders during COVID-19. Patients with pneumonia may develop thromboembolism, which may worsen the clinical course of the SARS-CoV-2 infection [8,9].

Treatment methods for patients with COVID-19-associated pneumonia include oxygen therapy and antiviral, anti-inflammatory, and immunosuppressive drugs depending on the phase of the disease, while low molecular weight heparin is recommended regardless of the stage of the illness as prophylaxis of thromboembolism [10,11,12]. One of the mechanisms of coagulopathy in COVID-19 is endothelial dysfunction leading to damage to the vessels in organs since the vascular endothelium is among the most critical targets of the virus [13].

The presence of the genetic material of SARS-CoV-2 in the retinal tissue has been confirmed postmortem by PCR [14]. The vascular endothelium plays a role in controlling vascular tone and maintaining the blood–retinal barrier. Its dysfunction can lead to microvascular ischemia due to vasoconstriction and inflammation [15].

The most current method to visualize macular micro vessels is by using non-invasive optical coherence tomography angiography (OCTA). The OCTA can successfully visualize the microvascular network of the retina with its blood flow in numerous eye disorders and general diseases [16]. Both OCT and OCTA are still getting improved with new, automated algorithms being introduced [17,18,19,20,21]. This helps both unexperienced ophthalmologists and enables more reliable comparison between studies.

The current study aimed to evaluate the microvascular changes in the retina based on OCTA in COVID-19 patients hospitalized due to bilateral pneumonia caused by SARS-CoV-2. The impact of comorbidities and the received treatment was also analyzed.

## 2. Materials and Methods

### 2.1. Subjects

A cross-sectional, consecutive, prospective case-control series analysis was carried out. Cases were selected from a population of COVID-19 patients with bilateral pneumonia hospitalized in the Department of Infectious Diseases of Municipal Hospital in Kielce during the pandemic spring wave caused by B.1.1.7 variant of SARS-CoV-2 from March to May 2021. The project was approved by the Bioethics Committee of Collegium Medicum of Jan Kochanowski University in Kielce (study code 54 approved on 1 July 2021).

Exclusion criteria related to eye diseases included as follows: myopia > 3 diopters, hyperopia > 3 diopters, retinal vascular disease, macular and optic nerve disease, previous ocular surgery including cataract surgery, glaucoma surgery, and other types of eye surgery, uveitis, ocular trauma, age-related macular degeneration, and other macular degenerations and media opacity affecting OCTA’s scan or image quality, diabetes mellitus. All examinations were performed by a single non-masked investigator, with standard protocol applied to each patient.

All patients signed the written informed consent to participate in the current study and underwent ophthalmological evaluation eight weeks after hospital discharge.

### 2.2. Characteristics of the Studied Group

The analyzed group consisted of 62 patients with male predominance (n = 42, 68%), the mean (M) ± standard error of the mean (SEM) age was 51.3 ± 1.4 years, and BMI M ± SEM was 28.5 ± 0.5 kg/m^2^. Thirty-nine patients suffered from comorbidities with the most common arterial hypertension (20 patients) followed by other cardiovascular diseases, including ischemic, valvular heart disease, and cardiac rhythm disturbances, which affected six patients. Five individuals had liver steatosis, and five had a history of malignant neoplasm (inactive). Data on the course of hospitalization were obtained retrospectively from hospital records. At the time of admission to the hospital, due to COVID-19, all patients were symptomatic. In all patients, the clinical diagnosis of COVID-19 was confirmed by the positive result of the real-time reverse transcriptase-polymerase chain reaction (RT-PCR) from nasopharyngeal swabs. The diagnosis of bilateral pneumonia was supported by typical chest computed tomography scan changes described in 26 patients.

Twenty-two patients were classified at baseline as stable with oxygen saturation (OS) >95%, twenty-nine were unstable with OS 91–95%, and the remaining eleven patients were assessed as unstable with OS ≤ 90%. The need for continuous low-flow oxygen therapy was documented in 23 patients. The most frequent drug used for the in-hospital treatment of COVID-19 was low-molecular-weight heparin in prophylactic dose, which was administered in fifty-nine patients; two individuals received a therapeutic dose. The antiviral agent remdesivir was used in 26 patients. Immunosuppressive treatment with dexamethasone was administered to 22 patients, while 3 received tocilizumab in a single dose of 600–800 mg, depending on the patient’s weight.

### 2.3. Treatment of COVID-19 Patients during Hospitalization

The low-molecular-weight heparin in prophylactic dose was administered in 59 COVID-19 patients for an average of 9 (6.25–12) days. Dexamethasone was administered in 22 infected patients, tocilizumab was administered in 3 patients in a single dose of 600–800 mg depending on the patient’s weight. Remdesivir was administered to 26 patients. The need for continuous oxygen therapy was in 23 patients for an average of 5 (4–10) days. Twenty-three patients received oxygen therapy during hospitalization for an average of 5 (4–10) days.

### 2.4. Ocular Characteristic of COVID-19 Patients

The COVID-19 patients (62 patients, number of eyes = 119) underwent complete ophthalmic examination, including a best corrected visual acuity (BCVA) test, in a LogMAR scale, intraocular pressure (IOP) measurement, a slit-lamp examination, OCT of the macula and optic disc and angio-OCT (OCTA).

The mean LogMar BCVA was 0.0 and the mean LogMar Reading Vision (RV) was 0.3, the spherical equivalent (SE) was 0.13 (0.13) D, the mean axial length was 23.55 (0.08) mm (Table 1).

### 2.5. Optical Coherence Angiography Measurements

All scans were acquired with Swept Source DRI-OCT Triton SS-OCT Angio (Topcon Inc., Tokyo, Japan). OCT protocols included 3D macula 7 × 7 mm scanning protocols, 3D Disc- 6 × 6 mm scanning protocols, and OCTA images were captured using the 4.5 × 4.5 mm and the 6 × 6 mm scanning protocols.

Structural OCT macular parameters were measured using the early treatment diabetic retinopathy (ETDRS) grid, centered in the fovea by manual fixation. Three areas of interest were defined as the fovea, inner ring (IR), and outer ring (OR). IR and OR include superior (S), inferior (I), nasal (N), and temporal (T) areas. The retinal stratus and parameters analyzed were total retina, retinal nerve fiber layer (RNFL), ganglion cell layer (GCL), and choroid thickness as delineated by the boundaries automatically defined by the built-in segmentation software. Optic nerve head RNFL thickness was measured in each quadrant using a radial scan centered on the optic nerve head and presented as mean RNFL.

The evaluated OCTA parameters were: vessel density (VD) in the three different plexi: superficial capillary plexus (SCP), deep capillary plexus (DCP), and choriocapillaris (CC) using the ETDRS grid subfields to define the areas of interest. Mean vessel density (VD) was calculated as the average value obtained in the parafoveal area, defined as the area conformed by the superior (S), inner nasal (N), inner inferior (I), and inner temporal (T) ETDRS subfields centered on the macula by fixation. The foveal avascular zone (FAZ) area was manually delineated on the SCP and the DCP by two independent graders, encompassing the central fovea where no clear and demarcated vessels were seen on the OCTA.

### 2.6. Statistical Analysis

Counts (n) and percentages (%) attendance were counted for all qualitative parameters in the COVID-19 patient group. Three groups of patients with saturation were distinguished using the following cut-off values: ≤90%, ≤95%, and >95%. In the distinguished groups, the distributions of the variables were checked with the quantitative Shapiro–Wilk test. The mean ($\bar{x}$) and standard error of the mean (SEM) were counted in the separate saturation groups. The difference between the mean and one-way analysis of variance (ANOVA) or Kruskal–Wallis test were used to assess differences between means. When significant differences were observed, the following tests were applied post-hoc: Bonferroni test or Dunn-Bonferroni test depending on the analysis applied. In order to determine the relationship between the parameter’s ocular and saturation, two types of correlation, Pearson or Spearman rank correlation, were considered. The following values were considered significant. The test values for which *p* ≤ 0.05 were considered significant. The statistical analysis was performed using the STATISTICA 13.3 statistical package (STATSOFT, PL, Kraków, Poland).

## 3. Results

A total of 119 eyes were included in the study group. In all, 42 men and 20 women with COVID-19 bilateral pneumonia participated in the analysis. The mean age of participants was 51.33 (SEM = 1.45).

The clinical course of SARS-CoV-2 disease was assessed with the ordinal scale based on the WHO recommendation, modified to an 8-score version to fit the specificity of the Polish healthcare system and used in previous SARSTer studies [4,15,16]. The scores were given at baseline and after 7, 14, and 28 days of hospitalization and were defined as follows: 1—not hospitalized, no activity restrictions; 2—not hospitalized, no activity restriction and/or requiring oxygen supplementation at home; 3—hospitalized, does not require oxygen supplementation and does not require medical care; 4—hospitalized, requiring no oxygen supplementation, but requiring medical care; 5—hospitalized, requiring normal oxygen supplementation; 6—hospitalized, on non-invasive ventilation with high-flow oxygen equipment; 7—hospitalized, for invasive mechanical ventilation or extracorporeal membrane oxygenation; 8—death. Improvement in the clinical course of COVID-19 was defined as a reduction in the score of at least 2 points [4]. COVID-19 patients participating in our study belonged to group 6.

### Structural OCT Outcomes Depending on Oxygen Saturation

The COVID-19 patients were divided in three groups depending on oxygen saturation: ≤90% (group 1), ≤95% (group 2), >95% (group 3).

There were no statistically significant differences between the different OCT parameters in COVID-19 patients according to oxygen saturation [≤90% (group 1), ≤95% (group 2), >95% (group 3)]. Group 1 consisted of 10 patients, including 19 eyes, group 2 consisted of 29 patients, including 56 eyes, and group 3 consisted of 23 patients, including 44 eyes (Table S1). There was a statistically significant decrease in retinal thickness [r = 0.50, *p* = 0.029 in inner, superior ring (ISR), r = 0.49, *p* = 0.034 in inner, temporal ring (ITR), r = 0.56, *p* = 0.012 in outer superior ring (OSR), r = 0.062, *p* = 0.004 in outer temporal ring (OTR)] in some areas of the macula in the group of patients with SpO2 equal or lower than 90%. A statistically significant correlation was found between choroidal thickness (BMCSI) and SpO2. For SpO2 equal to or lower than 90% r = 0.52, *p* = 0.021 in the outer nasal ring (ONR) and for the oxygen saturation of 90–95%, r = −0.38, *p* = 0.007 in the outer nasal ring (ONR).

A statistically significant negative correlation was reported between SpO2 ≤ 90% and RNFL optic disc (r = −0.65, *p* = 0.005 in superior RNFL optic disc, r = −0.60, *p* = 0.012 in temporal RNFL optic disc). A decrease in RNFL retinal thickness was observed in some areas of the central retina in COVID-19 patients with SpO2 ≤ 90% (r = 0.50, *p* = 0.032). As SpO2 ≤ 90% level, a decrease in macular GCL thickness was observed (r = 0.053, *p* = 0.02 in GCL inner superior ring (ISR), r = 0.46, *p* = 0.047 in GCL inner inferior ring (IIR). There was a positive statistically significant correlation between GCL in the outer inferior ring (OIR) and SpO2 of 90–95% (r = 0.33, *p* = 0.012). As the SpO2 decreased, the FAZ area in the SCP statistically significantly increased (r = −0,20, *p* = 0.025).

There was a statistically significant correlation between foveal VD (vessel density) in SCP and SpO2 (r = −0.22, *p* = 0.016). There was a statistically significant correlation between mean VD in DCP and SpO2 ≤ 90% (r = −0.55, *p* = 0.016) and between the temporal area of VD in DCP and SpO2 ≤ 90% (r = −0.47, *p* = 0.045).

There was a statistically significant correlation between the temporal area of VD in CC and SpO2 equal to or lower than 90% (r = −0.59, *p* = 0.007) (Table S2).

## 4. Discussion

In this study, we present the correlations between blood saturation and ocular parameters based on changes in blood flow observed in OCTA examination in COVID-19 patients. This is intended to clarify the role of hypoxia in metabolism and microvasculature of the retina and choroid.

Hypoxia and inflammation are linked at the molecular, cellular, and clinical levels. Factors that induce acute hypoxemia, such as SARS-CoV-2 disease enhance various cytotoxic functions of neutrophils and may stimulate hyperinflammation. Animal models showed that exposure to low oxygen concentrations results in increased vascular permeability, accumulation of inflammatory cells, and increased serum cytokine levels. Therefore, hypoxia is not only a consequence of respiratory disease but also contributes significantly to progressive lung damage and failure of other organs [22,23].

OCTA has revolutionized ophthalmic clinical practice. It produces high-contrast images of the retinal blood flow, with sufficiently high resolution to show the location of individual capillaries in the retina. OCTA can differentiate the SCP from the DCP and show how each plexus is affected in retinal vascular disease [14]. The OCT and OCTA are valuable tools in the objective estimation of retinal microvasculature involvement in COVID-19 patients in vivo, and obtained parameters can be used as potential biomarkers of vascular damage in other organs [12,24,25,26].

### 4.1. Retinal and Choroidal Changes in the Macular Region

We observed a significant thinning of retinal thickness in some macular areas in patients with SpO2 equal to or lower than 90%. A statistically significant correlation was found between choroidal thickness (BMCSI) and SpO2; we reported significant thinning of choroidal thickness in patients with SpO2 equal to or lower than 90%, but the thickness of choroid was correlated significantly negative in patients with SpO2 of 90–95%.

Thinning of the GCC on OCT may reflect the selective vulnerability of the inner retina to hypoxia, and secondary peripapillary edema development, likely as a result of post hypoxic inflammation [27]. The electrophysiologic measurements at high altitudes have shown changes suggesting the altered function of the inner and outer retina [28,29] with the retinal ganglion cells in the inner retina seeming to be particularly susceptible to transient hypoxia [30]. Human OCT studies relieved increased thickness of the retinal nerve fiber layer (RNFL) and ganglion cell layer (GCL) after ascent to high altitude, with lower in-air concentration [31,32]. Nickel et al. found the commonness of retinal vascular changes after 3-week hypoxia [33]. A significant decrease in total retinal thickness (TRT) in the induced systemic hypoxia mouse model was observed in a study by Mesentier-Louro et al. with significant thinning of the inner retina in the ganglion cell complex (GCC), but not outer retinal layers in the early stage of hypoxia. In contrast, after 18 h, thickening of the TRT was noticed relative to the 1 h group (*p* = 0.0002) and the baseline (*p* = 0.0320). The OCT features were only significant in the inner retina in the GCC (*p* < 0.0001) but not in the outer retina.

We also observed a significant thickening of RNFL in the optic disc, and significant thinning of RNFL in some macular areas in patients with SpO2 equal to or lower than 90%.

In a pig model of acute respiratory distress syndrome, RNFL thickness was increased and there was immunostaining for reactive oxygen species HIF-1 alpha and VEGF-A in retinal arterioles, suggestive of increased retinal vascular permeability and endothelial dysfunction [34]. In our study, the thinning of the RNFL in the macular area can be probably explained by prolonged hypoxia followed by increased retinal cell death.

### 4.2. FAZ Enlargement in COVID-19 Patients

We observed a significant increase in the size of the FAZ area in patients with lower values of SpO2. This area allows the most distinct vision because of the high cone density and absence of blood vessels. The circulation is particularly vulnerable in the FAZ, as in this region retinal blood vessels are absent. Therefore, the retinal cones of the FAZ area are completely dependent upon oxygen and nutrient delivery from the underlying choriocapillaris. The FAZ is therefore highly sensitive to ischemic events, and because of this, can act as an indicator of several pathological processes [14,35]. Enlargement of the FAZ area has been associated with ischemia in diabetic retinopathy and retinal vein occlusion [36]. A recent OCTA study excluded the correlation between FAZ size and duration of diabetes [35]. The noninvasive visualization of the fovea capillary network with advanced optical imaging facilitated a better understanding relationship between the FAZ and foveal pit morphology in different systemic diseases such as diabetes mellitus and other vascular diseases [13].

### 4.3. Choroidal Vessel Density Changes

In our study, we observed significantly greater vessel density in some areas in SCP, DCP, and CC in patients with SpO2 equal to or lower than 90%. The inner retina is supplied by the central retinal artery, but the outer avascular retina, which consists entirely of photoreceptors and Muller cell processes is supplied and nourished by choroid [37].

The influence of hypoxia on retinal and choroidal microcirculation was widely studied previously [38,39]. Because the retinal and choroidal circulations behave differently while responding to systemic hypoxia, the PaO_2_ (oxygen partial pressure) in the outer and inner retina behaves differently as well. The choroidal circulation does not seem to adapt to the metabolic needs of the outer retina and its resistance is not considered to change during hypoxia, although there are not many studies on the subject. Choroidal blood flow is likely to increase as a result of increased arterial pressure during hypoxia, but the choroidal circulation is regulated to some extent during moderate pressure changes, so this is not a major effect. As a result, when PaO_2_ falls, there is no mechanism to compensate and transport more blood to the choroid. In contrast to the choroid, the retinal circulation does regulate in response to metabolic demand, and blood flow goes up during hypoxia. It is sensible to ask whether hypoxia itself can result in neovascularization in the retina. In young animals, the retina shows elevated HIF-1alpha (hypoxia-inducible factor-1 alpha) and VEGF (vascular endothelial growth factor) before the development of the retinal vasculature, and this is most probably the main stimulating factor for the normal development of the retinal circulation. The elimination of HIF-1 alpha and VEGF in astrocytes does not change this, therefore the other cells or other compensatory processes must be involved. In the hypoxic conditions of oxygen-induced retinopathy, HIF-1 alpha and VEGF are also vital to abnormal neovascularization. Several hours of hypoxia in rats led to the upregulation of HIF-1 alpha protein and VEGF expression. Many researchers have suggested that the neovascularization in ischemic diseases such as diabetes or vein occlusion, which do not destroy the whole inner retina, may be caused by hypoxia, but the retinal disease always involves a complex mixture of events besides hypoxia, so the role of hypoxia itself is rarely certain [38].

In an examination of the diabetic patients without clinical retinopathy (NoDR) with OCT, significantly higher perfused capillary density (PCD) and the PCD were noted compared to the healthy control group. The PCD was more sensitive than FAZ metrics for detecting a difference between diabetic patients and the healthy group. The diabetic patients with NPDR (non-proliferative diabetic retinopathy) and PDR (proliferative DR) had progressively decreased PCD [40]. The increase was highest in the innermost 200-micrometer ring surrounding the FAZ, which is the region of highest metabolic demand in the retina. The impact of relative tissue hypoxia can be expected to be most evident in this area adjacent to the FAZ. These findings put forward that OCTA PCD may be a novel, more sensitive biomarker for detecting the earliest diabetic changes in patients with no other clinical features of diabetic retinopathy as well as objectively monitoring the clinical course of the disease. The increase in PCD observed in the NoDR group could be owing to new capillaries as in neovascularization, recruitment of reserve nonperfused capillary segments, or dilation of existing capillaries. Capillary dilation markedly increases volumetric retinal blood flow, which can be secondary to capillary dilation and could result in higher intravascular oxygen concentration in arterioles.

Relative tissue hypoxia can be an early trigger in the pathogenesis of the diabetic microvascular disease. Direct evidence of inner retinal hypoxia in diabetic cats without retinopathy has been observed using an intraretinal electrode. The tissue demand for oxygen is increased due to the need to accommodate increased levels of glucose. Relative hypoxia can occur as a result of reduced oxygen extraction from blood vessels by retinal tissues [9]. It is known that the retinal neurovascular unit reacts to tissue hypoxia (decreased pO2) and decreased pH (from increased lactic acid levels produced by anaerobic metabolism) with a functional autoregulatory dilation of the capillaries [41]. Reactive oxygen species (ROS) and the inflammatory milieu in diabetes likely contribute to functional capillary dilation, besides causing increased permeability, leukocyte stasis, and adhesion [41,42].

Another mechanism was proposed for increased retinal flow in well-controlled type 1 diabetics without retinopathy, reported on the absence of retinal arterial constriction in response to a pressure stimulus, attributing it to a defect in the myogenic response of the smooth muscle cells of retinal arterioles in the setting of diabetes [43]. A more permanent structural dilation may occur with prolonged dilation and endothelial cell proliferation in the setting of continued hypoxia. Increased blood flow through the dilated retinal capillaries would result in damage to the endothelial cells. With concomitant pericyte loss and formation of advanced glycation end-products, it would lead to the compromise of the microvascular wall integrity, causing recognizable features of diabetic retinopathy [44].

In our study, twenty-three patients were treated with continuous oxygen therapy for an average of 5 (4–10) days. The influence of hyperoxia on retinal and choroidal microvasculature was previously studied. When the arterial pO2 (oxygen tension) increases as a result of increased inspired O_2_, the changes in pO2 are opposite to those in hypoxia [38]. The choroidal circulation does not constrict during hyperoxia, PC (choriocapillaris pO2) increases proportionately with the increase in PaO_2,_ which was observed in several studies in various animal models. The amount of O_2_ transported by the choroid does not change much beyond the point of full hemoglobin saturation, but the increase in PC is quite essential because this is the driving force for O_2_ diffusion into the retina. If the increase in PC is large enough, O_2_ will diffuse to the inner retina. The value of PC will increase still further with hyperbaric oxygen. Therefore, hyperoxia and hyperbaric treatment for vascular occlusive disease have been suggested, but there is a misconception that hyperbaric O_2_ would be much better than 100% O_2_ at atmospheric pressure. Because the influence of hyperoxia on PC is large, the PO_2_ in the outer retina increases dramatically. The retinal circulation vessels constrict during hyperoxia. This tends to keep inner retinal PO_2_ close to levels during air-breathing, but the regulation is not perfect, because the diffusion of O_2_ from the choroid is unavoidable. The rise in inner retinal PO_2_ in animal models is considerably less than the increase in PO_2_ in the outer retina. Hypercapnia superimposed on hyperoxia can become smaller or eliminate the hyperoxic vasoconstriction of the retinal circulation and lead to much larger increases in inner retinal PO_2_ [38].

### 4.4. Tocilizumab Treatment Potential Impact on Retinal Microcirculation

Significantly decreased SpO2 ≤ 90% was one of the indications for the implementation of tocilizumab (TOC), apart from elevated interleukin 6 (IL-6) levels > 100 pg/mL and the need for oxygen supplementation. In these indications, the effectiveness of TOC was the best. TOC was used in three patients in our study group. This medication is aimed at blocking an IL-6 proinflammatory pathway. Despite the direct viral effect, the pathogenesis of COVID-19 includes an overproduction of cytokines [45]. This mechanism leads to systemic inflammation and hypoxia. The cytokine storm is responsible for organ failure and is sometimes the leading cause of death due to COVID-19 [46]. Therefore, TOC, which is a monoclonal antibody against IL-6 receptors, should be considered as a possible therapeutic option. Flisiak et al. observed that patients treated with TOC more frequently demonstrated a course of the disease with SpO2 (oxygen spirometry) below 90% at admission to the hospital (65.9%) compared to those without TOC (37.7%). Patients treated with TOC more often required normal- or high-flow oxygen supplementation (93.6%) compared to the non-TOC group (76.8%). These authors observed the highest reduction in mortality, the need for mechanical ventilation, and best clinical improvement at day 28 in patients receiving TOC with baseline IL-6 >100 pg/mL and SpO2 < 90% than in patients with SpO2 higher/equal to 90%. This observation might further underline that in patients with severe hypoxia, further deregulation between IL-6 levels and other cytokines is present, and possibly IL-6 activation is deeper and not counterbalanced by regulatory mechanism, which could explain the effect of TOC is more significant [45].

### 4.5. Limitations of the Study

Our study has several limitations. It could not be performed during the symptomatic, acute phase of COVID-19 due to the emergency condition and risk of contagion. Patients were burdened with additional diseases such as hypertension or dyslipidemia, so we cannot exclude the effect of these diseases. We have no baseline results of the OCT in COVID-19 patients before the disease, so we cannot compare these findings. The number of patients with SpO2 equal to or lower than 90% was small because some patients died or had a comorbid vascular disease that excluded them from the study. Most patients in our group had SpO2 above 90%.

A strong point is a group consisting of the selected patient without the influence of vascular disease such as diabetes mellitus. Only hospitalized COVID-19 patients were included in the study, forming a significant number of COVID-19 patients, examined at the same time point after discharge from the hospital.

## 5. Conclusions

This is the first study to describe the correlations between individual OCT parameters and SpO2 and to explain the effect of general hypoxia on ocular vascularization.

Our study demonstrated the effect of systemic hypoxia due to bilateral SARS-CoV-2 pneumonia on ocular parameters based on OCTA examination. Further follow-up of these patients is needed for long-term evaluation of the retina, choroid, and optic nerve for the development of degenerative changes resulting from systemic hypoxia.

## Figures and Tables

**Table 1:** Ocular characteristics of COVID-19 patients.

Variables	$\bar{x}$ (SEM)	Me (IQR)
**LogMar BCVA**	0.0 (0.0)	0.0 (0.00)
**LogMar Reading Vision**	0.3 (0.03)	0.3 (0.03)
**Spherical equivalent (D)**	0.13 (0.13)	0.0 (2.25)
**Axial length**	23.55 (0.08)	23.45 (1.05)

$\bar{x}$—mean, Me—median, SEM—standard error of mean; IQR—interquartile range; BCVA—best corrected visual acuity.

## Data Availability

All data can be obtained from the author, contact mail: kalmagda@gmail.com.

## Supplementary Materials

The following supporting information can be downloaded at: https://www.mdpi.com/article/10.3390/jpm12111824/s1. Table S1: Comparison of foveal (F) and parafoveal parameters of OCT parameters (RNFL RETINA, GCL, BMCSI, RETINAL THICKNESS, RNFL optic disc) and (optical coherence tomography) OCTA parameters; Table S2: The correlation between oxygen saturation (%) and foveal, parafoveal OCT parameters (RNFL RETINA, GCL, BMCSI, RETINAL THICKNESS, RNFL optic disc) and OCTA parameters.
