# Supplementary material for: The Effect of Reduced Oxygen Saturation on Retinal Microvascularization in COVID-19 Patients with Bilateral Pneumonia Based on Optical Coherence Tomography Study
Source: J Pers Med. 2022 Nov 2;12(11):1824. doi: 10.3390/jpm12111824 (PMC9692346; doi:10.3390/jpm12111824)
Supplement: Supplementary file 1 [file jpm-12-01824-s001.zip › Supplementary Table S1.pdf]

Supplementary Table S1. Comparison of foveal (F) and parafoveal parameters of OCT parameters (RNFL RETINA, GCL, BMCSI, RETINAL THICKNESS, RNFL optic disc) and (optical coherence tomography) OCTA parameters (SCP-superficial capillary plexus, DCP-deep capillary plexus, CC-choriocapillaris, FAZ s - superficial foveal avascular zone, FAZ d - deep foveal avascular zone, F- foveal area, S- superior area, N-nasal area, I-inferior area, T-temporal area, Mean SCP consist of S,N,I,T , Mean DCP consist of S,N,I,T areas , Mean CC consist of S,N,I,T areas, ISR-inner superior ring, IIR-inner inferior ring ,ITR- inner temporal ring, INR-inner nasal ring, OIR-outer inferior ring, OSR-outer superior ring, ONR- outer nasal ring, OTR- outer temporal ring) in COVID-19 patients depending on the level of oxygen saturation [≤90% (group 1), ≤95% (group 2), >95% (group3)]. Mean ±SEM (standard error of the mean) structural OCT values. Bold values denote statistical significance at the p<0,05 level.

**Variables**	Total	≤90 (n=10)	≤95(n=29)	>95 (n=23)	p
	$\bar{x}$ (SEM)	$\bar{x}$ (SEM)	$\bar{x}$ (SEM)	$\bar{x}$ (SEM)	
**OCT A_ F_ SCP**	20.71(0.39)	22.22(0.77)	20.97(0.54)	19.74(0.70)	0.083
OCT A_F_DCP	17.22(0.43)	18.55(0.77)	16.41(0.50)	17.68(0.81)	0.169
OCT A_F_CC	51.46(0.38)	52.02(0.98)	51.99(0.53)	50.55(0.65)	0.190
OCT A_S_SCP	48.27(0.25)	47.90(0.81)	48.19(0.35)	48.53(0.37)	0.834 K-W
OCT A_S_DCP	51.98(0.30)	52.28(0.82)	51.83(0.42)	52.05(0.51)	0.860
OCT A_S_CC	54.06(0.19)	53.92(0.51)	54.06(0.25)	54.13(0.35)	0.936
N_SCP (%)	45.11(0.23)	44.32(0.61)	45.38(0.33)	45.10(0.35)	0.277
N_DCP (%)	49.03(0.27)	48.68(0.60)	49.13(0.40)	49.05(0.48)	0.856
N_CC (%)	53.60(0.19)	53.83(0.43)	53.60(0.31)	53.50(0.26)	0.852
I_SCP (%)	47.20(0.40)	46.63(1.02)	47.50(0.40)	47.06(0.86)	0.734
I_DCP (%)	51.31(0.36)	51.35(0.93)	51.66(0.54)	50.85(0.59)	0.599
I_CC (%)	54.35(0.20)	54.39(0.49)	54.64(0.28)	53.95(0.36)	0.304 K-W
T_SCP (%)	46.65(0.20)	46.24(0.56)	46.59(0.29)	46.91(0.33)	0.728
T_DCP (%)	47.60(0.23)	48.39(0.45)	47.46(0.33)	47.43(0.43)	0.340
T_CC (%)	53.98(0.17)	54.34(0.49)	54.02(0.27)	53.76(0.24)	0.304
Mean SCP (%)	51.97(0.22)	51.83(0.60)	52.20(0.25)	51.75(0.42)	0.494 K-W
Mean DCP (%)	54.29(0.24)	54.81(0.56)	54.13(0.31)	54.26(0.45)	0.607
Mean CC (%)	66.86(0.12)	67.12(0.35)	67.08(0.15)	66.48(0.22)	0.072 K-W
FAZs (μm2)	326.27(9.84)	281.34(14.60)	329.38(15.56)	341.72(16.12)	0.117
FAZd (μm2)	354.37(12.83)	337.68(26.07)	334.19(16.46)	387.27(24.87)	0.145
RNFL OPTIC DISC_S	132.67(1.33)	137.59(1.76)	133.09(1.85)	130.23(2.52)	0.122 K-W
RNFL OPTIC DISC_N	83.38(1.32)	86.00(3.57)	80.79(1.79)	85.66(2.24)	0.169
RNFL OPTIC DISC_I	135.33(1.62)	131.94(6.97)	135.57(2.06)	136.34(2.16)	0.920 K-W
RNFL OPTIC DISC_T	77.11(4.28)	68.12(2.19)	82.86(8.81)	73.27(1.49)	0.087 K-W
Total thickness RNFL OPTIC DISC	106.25(0.90)	107.29(1.57)	105.88(1.31)	106.32(1.61)	0.829 K-W
RETINAL THICKNESS F foveal	245.29(2.32)	244.42(4.04)	248.96(2.93)	240.98(4.72)	0.619 K-W
RETINAL THICKNESS ISR_inner superior ring	318.46(1.50)	320.79(2.54)	318.13(2.36)	317.89(2.54)	0.859 K-W
RETINAL THICKNESS INR inner nasal ring	317.82(1.63)	320.58(2.58)	317.71(2.46)	316.77(2.92)	0.778 K-W
RETINAL THICKNESS IIR_inner inferior ring	315.33(1.47)	315.79(2.40)	314.79(2.41)	315.82(2.35)	0.942
RETINAL THICKNESS ITR inner temporal ring	304.54(1.89)	308.79(2.46)	305.27(2.51)	301.77(3.84)	0.639 K-W
RETINAL THICKNESS OSR outer superior ring	277.76(1.41)	278.37(2.00)	277.21(1.93)	278.20(2.80)	0.691 K-W
RETINAL THICKNESS ONR outer nasal ring	290.71(1.50)	292.58(2.37)	290.27(2.47)	290.45(2.42)	0.863

RETINAL THICKNESS OIR outer inferior ring	266.13(1.22)	266.21(1.84)	268.07(2.07)	263.64(1.76)	0.254 K-W
RETINAL THICKNESS OTR outer temporal ring	259.80(1.48)	264.42(1.90)	261.98(1.97)	255.02(2.90)	0.056 K-W
RNFL RETINA F	3.58(0.23)	3.26(0.42)	3.98(0.40)	3.20(0.29)	0.318 K-W/
RNFL RETINA ISR	29.15(0.24)	29.53(0.67)	28.91(0.33)	29.30(0.41)	0.617
RNFL RETINA INR	24.26(0.21)	24.11(0.42)	24.13(0.35)	24.50(0.32)	0.357 K-W
RNFL RETINA IIR	29.52(0.32)	28.89(0.48)	29.19(0.58)	30.25(0.40)	0.114 K-W
RNFL RETINA ITR	20.03(0.24)	20.47(0.57)	20.04(0.34)	19.84(0.43)	0.682
RNFL RETINA OSR	41.82(0.53)	41.89(1.14)	41.63(0.88)	42.05(0.77)	0.662 K-W
RNFL RETINA ONR	52.52(0.77)	51.74(1.73)	51.95(1.13)	53.59(1.30)	0.567
RNFL RETINA OIR	43.70(0.63)	43.00(0.93)	43.96(1.09)	43.66(0.90)	0.870
RNFL RETINA OTR	23.11(0.36)	23.63(0.54)	22.79(0.39)	23.30(0.81)	0.451 K-W
GCL F	50.61(1.07)	50.63(1.68)	52.38(1.91)	48.36(1.37)	0.561 K-W
GCL ISR	122.66(0.86)	122.37(1.51)	122.27(1.38)	123.30(1.40)	0.855 K-W
GCL INR	118.09(0.89)	117.63(1.50)	117.70(1.40)	118.80(1.48)	0.783
GCL IIR	121.63(1.33)	120.53(1.52)	119.91(2.52)	124.30(1.45)	0.328 K-W
GCL ITR	110.71(0.86)	111.37(1.35)	110.82(1.13)	110.27(1.74)	0.957 K-W
GCL OSR	106.42(0.85)	105.68(1.50)	106.61(1.44)	106.50(1.27)	0.927 K-W
GCL ONR	122.32(1.06)	120.53(1.85)	122.29(1.71)	123.14(1.69)	0.715
GCL OIR	105.64(0.91)	104.26(1.30)	107.02(1.59)	104.48(1.27)	0.362
GCL OTR	93.08(0.73)	94.05(1.14)	93.63(1.17)	91.95(1.22)	0.498
BMCSI F	267.10(6.51)	274.74(15.03)	269.23(9.05)	261.09(11.79)	0.750
BMCSI ISR	268.67(5.88)	279.53(10.88)	271.07(8.74)	260.93(10.38)	0.335 K-W
BMCSI INR	250.39(6.25)	253.63(14.82)	254.45(9.14)	243.82(10.62)	0.726 K-W
BMCSI IIR	257.39(6.43)	255.05(14.59)	260.70(9.40)	254.18(11.11)	0.890
BMCSI ITR	258.55(6.25)	259.05(12.62)	259.43(8.86)	257.20(11.56)	0.771 K-W
BMCSI OSR	266.07(5.44)	260.26(10.06)	266.59(7.64)	267.91(10.31)	0.894
BMCSI ONR	201.08(6.14)	196.95(13.50)	207.20(9.45)	195.09(9.96)	0.644
BMCSI OIR	240.55(6.41)	229.95(11.06)	245.61(10.20)	238.70(10.58)	0.688
BMCSI OTR	237.92(5.68)	232.21(10.49)	237.82(8.78)	240.52(9.67)	0.991 K-W

K-W- Kruskall-Wallis non-parametric analysis of variance
