# Supplementary material for: The Effect of Reduced Oxygen Saturation on Retinal Microvascularization in COVID-19 Patients with Bilateral Pneumonia Based on Optical Coherence Tomography Study
Source: J Pers Med. 2022 Nov 2;12(11):1824. doi: 10.3390/jpm12111824 (PMC9692346; doi:10.3390/jpm12111824)
Supplement: Supplementary file 1 [file jpm-12-01824-s001.zip › Supplementary Table S2.pdf]

Supplementary Table S2. The correlation between oxygen saturation (%) and foveal, parafoveal OCT parameters (RNFL RETINA, GCL, BMCSI, RETINAL THICKNESS, RNFL optic disc) and OCTA parameters (SCP-superficial capillary plexus, DCP-deep capillary plexus, CC-choriocapillaris, FAZ s – superficial foveal avascular zone, FAZ d- deep foveal avascular zone, F- foveal area, S- superior area, N-nasal area, I-inferior area, T-temporal area, Mean SCP consist of S,N,I,T , Mean DCP consist of S,N,I,T areas , Mean CC consist of S,N,I,T areas) in COVID-19 patients. Mean ±SEM (standard error of the mean) structural OCT values. Bold values denote statistical significance at the p<0,05 level.

**Variables**	**Oxygen saturation**	**p**
OCT A F SCP	**-0.22**	**0.016**[P]
OCT A F DCP(LOG10)	-0.13	0.171[P]
OCT A F CC	-0.11	0.215[SP]
OCT A S SCP	0.08	0.357[SP]
OCT A S DCP	-0.01	0.950[SP]
OCT A S CC	-0.01	0.896[SP]
N SCP (%)	0.11	0.208[SP]
N DCP (%)	0.01	0.943[P]
N CC (%)	-0.05	0.564[P]
I_SCP (%)	0.13	0.175[SP]
I DCP (%)	-0.08	0.373[SP]
I CC (%)	-0.08	0.351[P]
T SCP (%)	0.07	0.436[P]
T DCP (%)	-0.16	0.085[P]<br>(for saturation level ≤90%: r= -0.47; p=0.045)
T CC (%)	-0.12	0.181[SP]<br>(for saturation level ≤90%: r=-0.59;p=0.007)
Mean SCP (%)	0.02	0.867[SP]
Mean DCP (%)	-0.15	0.113[P](for saturation level ≤90%: r=-0.55;p=0.016)
Mean CC (%)	-0.14	0.115[SP]
FAZs (μm2)	**-0.20**	0.025[P]
FAZd (μm2)	0.14	0.138[SP]
RNFL OPTIC DISC S	-0.13	0.175[SP]<br>(for saturation level ≤90%: r= -0.65;p=0.005)
RNFL OPTIC DISC N	0.08	0.367[P]
RNFL OPTIC DISC I	0.02	0.802[SP]
RNFL OPTIC DISC T	0.10	0.262[SP]<br>(for saturation level ≤90%: r= -0.60;p=0.012)
Total thickness RNFL OPTIC DISC	-0.03	0.767[P]
F RETINAL THICKNESS	-0.07	0.474[SP]
RETINAL THICKNESS ISR	-0.06	0.537[SP]<br>(for saturation level ≤90 % r=0.50;p=0.029)
RETINAL THICKNESS INR	0.06	0.505[P]
RETINAL THICKNESS IIR	0.04	0.680[P]
RETINAL THICKNESS ITR	-0.09	0.309[SP]<br>(for saturation level ≤90 %: r=0.49;p=0.034)
RETINAL THICKNESS OSR	-0.05	0.579[SP]<br>(for saturation level ≤90%: 0.56;p=0.012)
RETINAL THICKNESS ONR	-0.07	0.457[SP]

RETINAL THICKNESS OIR	-0.02	0.816^{P}
RETINAL THICKNESS OTR	-0.07	0.446^{P}<br>(for saturation level ≤90 %: r=0.62;p=0.004)
RNFL RETINA F	-0.07	0.497^{Sp}
RNFL RETINA ISR	-0.01	0.904^{P}
RNFL RETINA INR	0.11	0.237^{Sp}
RNFL RETINA IIR	0.13	0.162^{Sp}<br>(for saturation level ≤ 90%; r=0.50; p=0.030)
RNFL RETINA ITR	-0.08	0.407^{Sp}<br>(for saturation level ≤90 %: r=0.50; p=0.032)
RNFL RETINA OSR	0.09	0.334^{Sp}
RNFL RETINA ONR	0.05	0.602^{P}
RNFL RETINA OIR	0.004	0.958^{P}
RNFL RETINA OTR	-0.02	0.826^{Sp}
GCL F	-0.11	0.224^{Sp}
GCL ISR	0.06	0.538^{Sp}<br>(for saturation level ≤ 90%: r=0.53;p=0.02)
GCL INR	0.07	0.456^{Sp}
GCL IIR	0.15	0.109^{Sp} (for saturation level ≤ 90%: r=0.46;p=0.047)
GCL ITR	0.01	0.955^{Sp}
GCL OSR	0.10	0.278 ^{Sp}
GCL ONR	0.08	0.386^{P}
GCL OIR	0.03	0.700^{P}<br>(for saturation level: 90%-95%; r=0.33; p=0.012)
GCL OTR	0.003	0.974^{P}
BMCSI F	-0.01	0.899^{P}
BMCSI ISR	-0.04	0.657^{P}
BMCSI INR	-0.02	0.862^{P}
BMCSI IIR	0.04	0.658^{P}
BMCSI ITR	0.03	0.720^{P}
BMCSI OSR_	0.09	0.328^{P}
BMCSI ONR	0.11	0.223^{P}<br>(for saturation level: ≤90%; r=0.52;p=0.021;<br>for saturation level: 90%-95%: r= -0.38;p=0.007)
BMCSI OIR	0.08	0.407^{P}
BMCSI OTR	0.08	0.365^{P}

^{Sp}-Spearman rank correlation, ^{P}- Pearson correlation
